# Methionine Restriction Differentially Modulates Expression of Genes in the Base Excision Repair Pathway in Rat Brain and Liver

**DOI:** 10.3390/biom15070969

**Published:** 2025-07-05

**Authors:** Ricardo Gredilla, Monica Lopez-Torres, Ines Sanchez-Roman

**Affiliations:** 1 Department of Physiology, Faculty of Medicine, Complutense University of Madrid (UCM), 28040 Madrid, Spain; gredilla@ucm.es; 2 Department of Genetics, Physiology and Microbiology, Faculty of Biological Sciences, Complutense University of Madrid (UCM), 28040 Madrid, Spain; mltorres@ucm.es

**Keywords:** aging, DNA repair, genome stability, oxidative stress, glycosylase, endonuclease, longevity

## Abstract

Methionine restriction (MetR) is a dietary intervention that extends mean and maximum life span in rodents, at least in part, by reducing oxidative stress and promoting DNA stability in different tissues. Regarding DNA stability, DNA repair pathways play a critical role, both in the nuclear and mitochondrial fractions. Base excision repair (BER) is the main one involved in the repair of oxidative damage, as well as the main one in mitochondria. Despite the relevance of DNA repair in DNA maintenance, it is not known whether MetR regulates BER as a mechanism of preserving genomic stability. In this study we analyzed, for the first time, the effect of 40% MetR for 7 weeks on BER in rat brain cortex and liver, focusing on the expression of several key BER genes. In the brain cortex, MetR significantly increased the gene expression of the DNA glycosylase *Ogg1* and the DNA endonuclease *Ape1* while reducing DNA *polymerase γ* gene expression. Conversely, MetR led to a general reduction in the expression of BER genes in the liver. Our findings highlight a tissue-specific regulation of the BER gene expression in response to MetR. Different potential mechanisms underlying these changes in BER, such as DNA methylation or activation of signaling pathways, are discussed.

## 1. Introduction

### 1.1. Aging and DNA Instability

Aging is a progressive and unavoidable process that nowadays constitutes a major health concern in most Western countries. In addition, aging is a multifactorial and complex process, with the increase in age-related DNA instability being one of the major factors that contributes to the physiological decline observed as animals age [1,2]. Interestingly, it has been known for decades that different dietary interventions can have a great impact on slowing aging and the onset of age-related diseases [3,4,5]. Since DNA instability is one of the factors that have been pointed out as important determinants of the aging process [1,2], an increasing number of studies have stressed the role of DNA repair mechanisms not only as essential in maintaining genome integrity but also as critical in the progression of physiological decline during aging [6,7,8]. DNA is constantly exposed to damaging agents, both endogenous and exogenous. DNA lesions generated by free radicals are highly mutagenic, and DNA repair mechanisms play a central role in preventing the accumulation of DNA mutations and in the maintenance of DNA stability. Both mitochondrial DNA (mtDNA) and nuclear DNA (nDNA) are exposed to such damage, and hence, repair mechanisms have evolved in both compartments in order to preserve DNA integrity [9,10].

Several major DNA repair pathways have been described in the nuclei [10,11]. However, it is considered that the main repair pathway for the mitochondrial genome is base excision repair (BER) [9]. Various proteins involved in other repair pathways than BER have also been reported to be present in mitochondria [12,13]. However, it remains to be established whether all those DNA repair routes are actually functional in mammalian mitochondria and how they are regulated. Interestingly, all DNA repair proteins involved in mtDNA repair are encoded in nDNA, and most of the proteins involved in nuclear and mitochondrial DNA repair are encoded by the same genes, with different splicing or protein processing [14,15].

**Figure 1:**
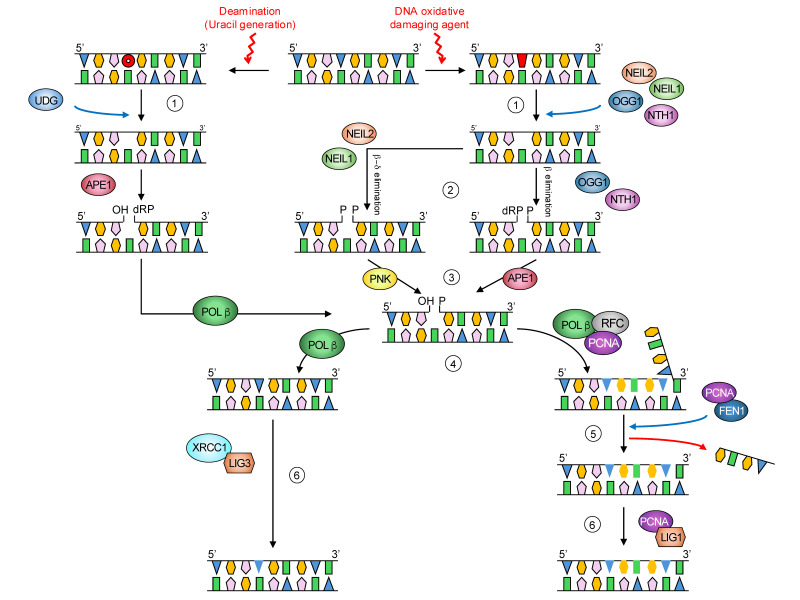
Base excision repair pathway: (1) The mammalian base excision repair pathway is initiated by removal of the modified base by a lesion-specific monofunctional (UDG) or bifunctional DNA glycosylase (NTH1, OGG1, NEIL1, or NEIL2). (2) After excision by monofunctional DNA glycosylases, the incision of the DNA backbone at the AP site is then processed by APE1. Bifunctional DNA glycosylases excise modified bases, followed by DNA backbone incision at the AP site via β- or βδ-elimination facilitated by the intrinsic AP lyase activity of these enzymes. (3) The 3′- and 5′-termini of the single-strand break are processed by DNA polymerase (POL) β, APE1, or PNKP depending on their specific nature. These are obstructive, and their removal generates a 3′-OH and 5′-P termini at the strand break, which is the normal substrate for DNA polymerases. (4) BER can then proceed through a long- or a short-patch subpathway. In the latter, single-nucleotide gap filling is performed by POLβ. Long-patch (LP) BER involves the incorporation of 2–12 nucleotides during repair synthesis by POLβ, interacting with other proteins such as PCNA and RFC. LP-BER results in the exposure of the ancient DNA strand as part of a single-strand overhang or a flap structure. (5) These flap structures are recognized and cleaved by flap endonuclease 1 (FEN1). POLβ is the only DNA polymerase depicted in the scheme, as it has been reported to be the main DNA polymerase in BER. However, other polymerases, such as POLγ in mitochondria, can also participate in the BER pathway. (6) Finally, ligation of the DNA backbone by DNA ligases (LIG1 or LIG3) interacting with other proteins (XRCC1 or PCNA) completes the BER pathway.

Among all the DNA repair pathways, BER is the main one involved in the recognition and removal of damage generated by oxidation, alkylation, and deamination processes. Deficiencies in this pathway have been associated with various cancers, neurodegeneration, and aging [9,16,17,18]. It is a four-step pathway with several proteins involved. Briefly, substrate-specific DNA glycosylases, such as uracil DNA glycosylase (UDG) or 8-oxoguanine DNA glycosylase (OGG1), are responsible for the recognition and removal of modified bases. They generate an abasic site, which is then processed by an apurinic/apyrimidinic endonuclease (APE1). Similarly to nuclear BER, mitochondrial BER (mtBER) can proceed through two different sub-pathways: short- or long-patch BER. The short-patch BER involves the incorporation of a single nucleotide into the gap by a DNA polymerase (POL), followed by strand ligation primarily by DNA ligase (LIG) 3. The long-patch BER involves the incorporation of several nucleotides, usually two to seven, by a DNA polymerase, followed by cleavage of the resulting 5′ flap by accessory proteins such as FEN-1 and ligation by LIG3 (Figure 1).

Until recently, the only known DNA polymerase in mammalian mitochondria was POLγ; however, additional polymerases have lately been identified in these organelles, POLβ being among them [19]. As mentioned previously, different studies have stressed the critical role that DNA repair mechanisms play in health and disease [20,21,22,23]. Thus, experimental approaches that improve health status or those that reduce the progression of aging and age-related diseases might exert their beneficial effects through modulation of DNA repair mechanisms.

### 1.2. Anti-Aging Dietary Interventions

Dietary interventions that can modulate several of the proposed hallmarks of aging [1,2] have become interesting approaches to the study of aging and longevity. During the last several years, different strategies have been used to improve aging and postpone the onset of age-related disorders. Among them, a variety of drugs/supplements that promote healthspan and/or life extension in nutritional interventions have become very popular. However, investigations on how dietary interventions affect DNA repair are scarce. The most robust results have been recently reported regarding interventions affecting NAD+ levels, one of the most extensively used supplements in the aging research field. NAD+ is an essential metabolite involved in multiple reactions associated with most of the pathways related to the hallmarks of aging [24]. Specifically, NAD+-dependent processes are involved in genome maintenance and DNA repair mechanisms. Aging is characterized by reduced NAD+ levels that impair DNA repair and increase DNA damage, which can contribute to an accumulation of mutations and cellular dysfunctions generally associated with aging [25]. The pharmacological upregulation of cellular NAD+ via NAD+ precursors, such as the use of NR (nicotinamide riboside), NMN (nicotinamide mononucleotide), or NAM (nicotinamide), are in the current scientific spotlight because of its beneficial effects on age-related diseases (reviewed in [24]) mediated at least partly by NAD+ involvement in the activation of BER pathways to maintain genome stability. Thus, it has been shown that dietary supplementation with NAD+ precursors in an Alzheimer’s disease (AD) animal model with DNA repair deficiency and in AD patients’ fibroblasts reduces DNA damage and apoptosis, likely through the enhancement of DNA damage response [26]. Moreover, the restitution of intracellular NAD+ levels in Ataxia telangiectasia animal models stimulates DNA repair [27].

Caloric restriction (CR), avoiding undernutrition, continues to be the best experimental manipulation capable of decreasing the aging rate and increasing mean and maximum lifespan in a wide range of organisms [4,28,29]. The life extension effect of CR can reach up to 50% in rodents, and it has been observed not only when initiated at a young age but also when implemented at middle age [30,31,32]. Besides its effect on longevity, CR also attenuates the incidence and progression of many age-related pathologies, including cardiomyopathy, nephropathy, diabetes, hypertension-related diseases, autoimmune diseases, and several neurodegenerative disorders like Parkinson’s or Alzheimer’s disease [33,34,35]. Although the longevity extension effect is controversial when CR has been investigated in rhesus monkeys, important beneficial effects on different age-related diseases have been reported [36,37]. Benefits for human health of CR have also been reported to reduce obesity, insulin resistance, atherosclerosis, inflammation, and oxidative stress [38].

It has been extensively reported that CR decreases oxidative stress [39,40,41]. Research in rodents has shown that long-term 40% CR significantly decreases mitochondrial reactive oxygen species (mtROS) generation in different rat tissues [40,41,42,43,44] as well as in mice [45,46]. In agreement with the decrease in mtROS production, CR also reduced oxidative damage to mtDNA alone, or both mtDNA and nDNA, depending upon the organ studied [40,41,42,44]. The longevity extension effect of CR was initially attributed to a decreased calorie intake rather than to decreases in specific dietary components. Studies questioned this classical consensus [47,48] and a significant number of investigations have reported increases in maximum longevity after protein restriction (PR) in rats or mice (reviewed in [49]), although to a lesser extent than CR. Furthermore, the restriction of a single amino acid, methionine (MetR), increases longevity in rats [50,51] and mice [52,53] to the same extent as PR. Similar results have been reported in yeast, *D. melanogaster*, and *C. elegans* [54,55,56]. In addition to the life extension effect, MetR also decreases the incidence of age-associated degenerative diseases [52,57,58,59]. Isocaloric MetR (80% and 40%) lowers mtROS production, mainly at complex I, and oxidative damage to mtDNA is measured as 8-oxodG levels in various tissues [60,61]. These results suggest that the reduced ingestion of a single molecule, methionine, during MetR, PR, and CR can be responsible for the decreases in mtROS production and mitochondrial oxidative stress reported in these dietary interventions. Although CR appears to exert a stronger lifespan extension effect than MetR, it is associated with significant reductions in body weight, which are not observed after MetR [61]. Moreover, although DR has been reported to promote healthy aging in human volunteers [62], it would not be a realistic public health intervention because of poor compliance with even mild dietary restriction regimes [63]. Thus, despite a less pronounced effect in lifespan extension, MetR might be a potentially more realistic intervention since it implies the reduction in a specific nutritional component of the diet. While DNA repair mechanisms have been studied in different animal models subjected to various degrees of CR [64,65,66], the potential role of MetR in regulating those mechanisms has not been explored to date. Thus, high DNA repair could contribute to the lower oxidative damage observed in methionine-restricted animals and to the lifespan extension effect of this dietary intervention.

The aim of the present study was to analyze the effect of methionine restriction on the gene expression of key enzymes of the BER pathway. To this end, the levels of mRNA of different BER enzymes were quantified in brain and liver tissue from control and methionine-restricted rats. These two tissues are of particular interest due to their critical role in systemic physiology. Liver has a central role in metabolism, detoxification and homeostasis, and different reports have suggested that liver aging may have broader implications for systemic aging and age-related diseases [67,68]. On the other hand, the brain is crucial due to its implications for cognitive function, neuroplasticity, and its susceptibility to age-related neurodegenerative diseases. This investigation would help to better understand the mechanisms through which a nutritional intervention, such as methionine restriction, preserves hepatic and brain functionality, and, therefore, may retard the aging process.

## 2. Materials and Methods

### 2.1. Animals and Diets

Sixteen male Wistar rats of 200–250 g of body weight (2 months old) purchased from Harlan Laboratories (Rossdorf, Germany) were caged individually and maintained with *ad* *libitum* access to food and water in a 12:12 (light/dark) cycle at 22 ± 2 °C and 50 ± 10% relative humidity at the facilities of the Complutense University animal house (ANUC ES28079000086). All the procedures were performed in accordance with institutional guidelines and were approved by the Experimental Animal Committee from the UCM/CAM. Semi-purified diets prepared by MP Biochemicals (Irvine, CA, USA) and imported to Spain by Leti (Barcelona, Spain) were used. The detailed diet composition is shown in Table 1. The composition of the 40% MetR diet was similar to that of the control diet, except that L-methionine was present at 0.516%, which corresponds to an amount of this amino acid 40% lower than in the control diet (0.86%). The 0.34% absolute decrease in L-methionine in the 40% MetR diet was compensated for by increasing all the other dietary components proportionally to their presence in the diet. Since the % absolute decrease in L-methionine was small, the % presence of all the other dietary components was almost the same in the two experimental diets. Both diets were isocaloric, and the macronutrient composition was similar. Animals were weighed weekly. After 7 weeks of dietary treatment, the animals were sacrificed at 9 a.m. by decapitation, and liver and brain (frontal cortex) samples were obtained, flash frozen in liquid nitrogen, and stored at −80 °C for later processing.

### 2.2. RNA Isolation and RT-PCR

RNA was isolated from liver and brain (cortex) samples using TRIsure^TM^ (BIOLINE, London, UK), following the manufacturer’s protocol. The purity of the isolated RNA was estimated by 1.5% agarose gel electrophoresis, and RNA concentration was determined by spectrophotometry (Nanodrop 2000-thermoscientific). cDNA synthesis was performed by reverse transcription of 1 µg RNA using a High-Capacity cDNA Reverse Transcription Kit (Applied Biosystems, Thermo Fisher Scientific, Madrid, Spain). The resulting cDNA samples were used with specific primers (Table 2) and 1× Takara SYBR Green Premix Ex Taq (Takara BIO Inc., Otsu, Japan) to perform quantitative real-time PCR. RT-PCRs were run in a MyiQ™2 Two-Color Real-Time PCR Detection System (Bio-Rad laboratories, Hercules, CA, USA)) using the following conditions: 2 min at 50 °C, 10 min at 95 °C, followed by 40 cycles of 15 s at 95 °C and 1 min at 60 °C. Duplicates of each sample were included, and melting curves were performed in order to verify the specificity of the amplification. 18S rRNA was used as an endogenous control to normalize the results. This gene is widely used as a housekeeping gene in quantitative PCR studies due to its stable expression across a broad range of tissues and experimental conditions. Moreover, we have confirmed its low variability in previous investigations. This consistency makes it a reliable internal control for the normalization of gene expression data, enhancing the accuracy and reproducibility of qPCR results. Relative gene expression was determined by using the 2^−∆∆Ct^ method.

### 2.3. Statistical Analysis

Data are presented as mean ± SEM (Standard Error of Mean) or median ± IQR (Interquartile range). Statistical evaluation was performed using GraphPad PRISM (version 6.01, La Jolla, CA, USA). Normal distribution of variables was evaluated by the Shapiro–Wilk test, and nonparametric tests were used when appropriate. Comparison between two groups was performed by a *t*-test or Mann–Whitney U-test (in case of no normal distribution). *p*-values < 0.05 were considered statistically significant.

## 3. Results

### 3.1. Body Weight of Rats and Food Intake Were Not Affected by Methionine Restriction

**Figure 2:**
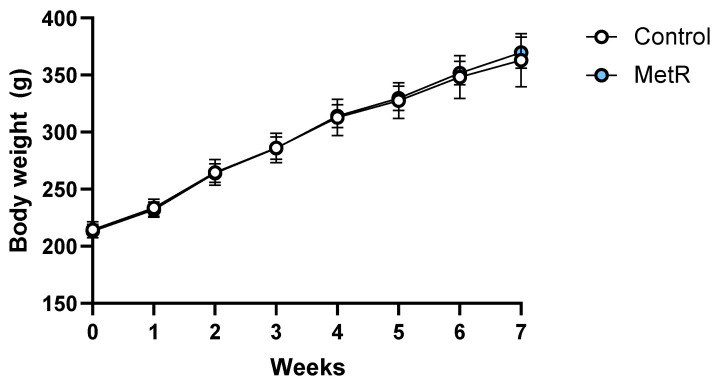
Body weight (g) over the course of the dietary intervention in the control (n = 8) and 40% methionine-restricted rats (MetR; n = 8). The results are expressed as the mean ± SEM.

Similarly to previous studies in our laboratory, the animals subjected to MetR did not significantly change their body weight compared to control animals after 7 weeks of treatment (Figure 2).

### 3.2. Rats Subjected to Methionine Restriction Show Similar Transcription Levels of Mnsod to Control Counterparts Both in Liver and Brain

**Figure 3:**
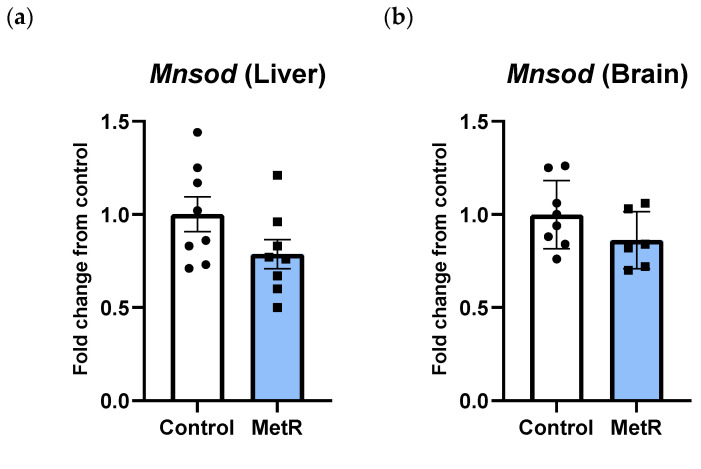
mRNA levels of *Mnsod* in the (**a**) liver and (**b**) brain of the control and 40% methionine-restricted rats (MetR). The results are expressed as the mean ± SEM relative to control animals; n = 8, except brain in MetR group (n = 6).

After methionine restriction, transcription levels of the mitochondrial isoform of superoxide dismutase (*Mnsod*) were reduced by 20% in the liver (Figure 3a) and by 16% in the brain (Figure 3b); however, the differences did not reach statistical significance in any of the tissues (*p* = 0.11 in liver and *p* = 0.16 in brain).

### 3.3. DNA Glycosylases and Ape1 Genes Were Downregulated by Methionine Restriction in Rat Liver

**Figure 4:**
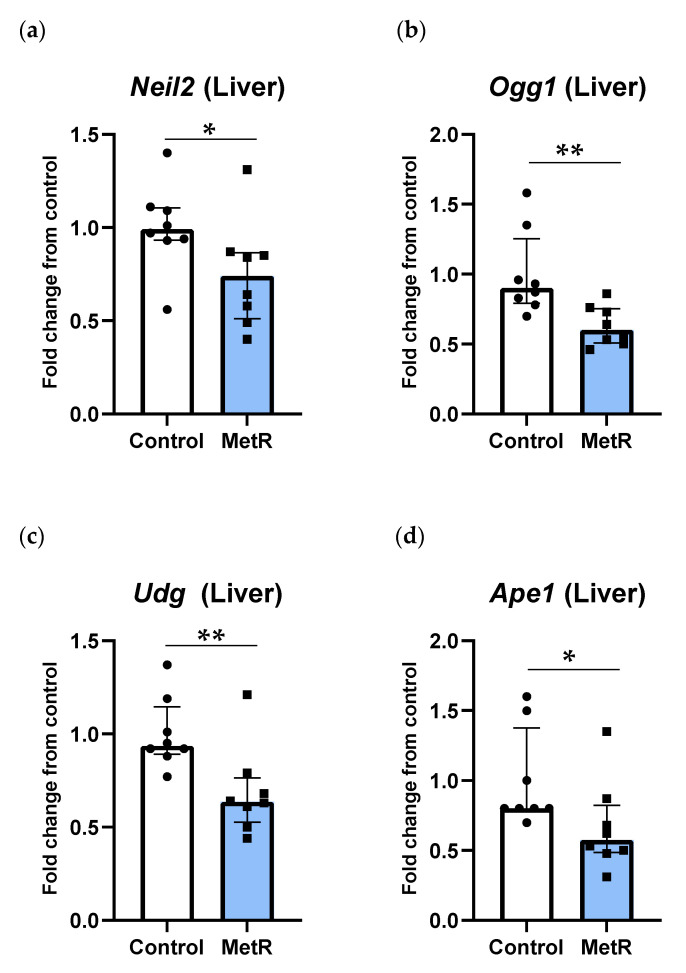
Hepatic mRNA levels of (**a**) *Neil2*, (**b**) *Ogg1*, (**c**) *Udg*, and (**d**) *Ape1* of the control (n = 8) and 40% methionine-restricted rats (MetR; n = 8). The results are expressed as the median ± IQR relative to control animals. * *p* ≤ 0.05; ** *p* ≤ 0.01.

After methionine restriction, the genes related to the first steps of the BER pathway were significantly downregulated (Figure 4). NEIL2 and OGG1 are DNA glycosylases related to the recognition and excision of oxidative damage in DNA. Transcription of both genes was significantly reduced after MetR. *Neil2*’s gene transcription was reduced by 25% (*p* = 0.037), while *Ogg1*’s underwent a nearly 40% reduction (*p* = 0.003). Similarly, transcription of the *Udg* gene, the main DNA glycosylase recognizing duracil generated by cytosine deamination, was significantly decreased by 30% (*p* = 0.009). In addition, *Ape1* gene expression was also significantly reduced by 30% after MetR (*p* = 0.024).

### 3.4. Transcription of Genes Related to DNA Polymerases but Not DNA Ligases Was Reduced After Methionine Restriction in Rat Liver

**Figure 5:**
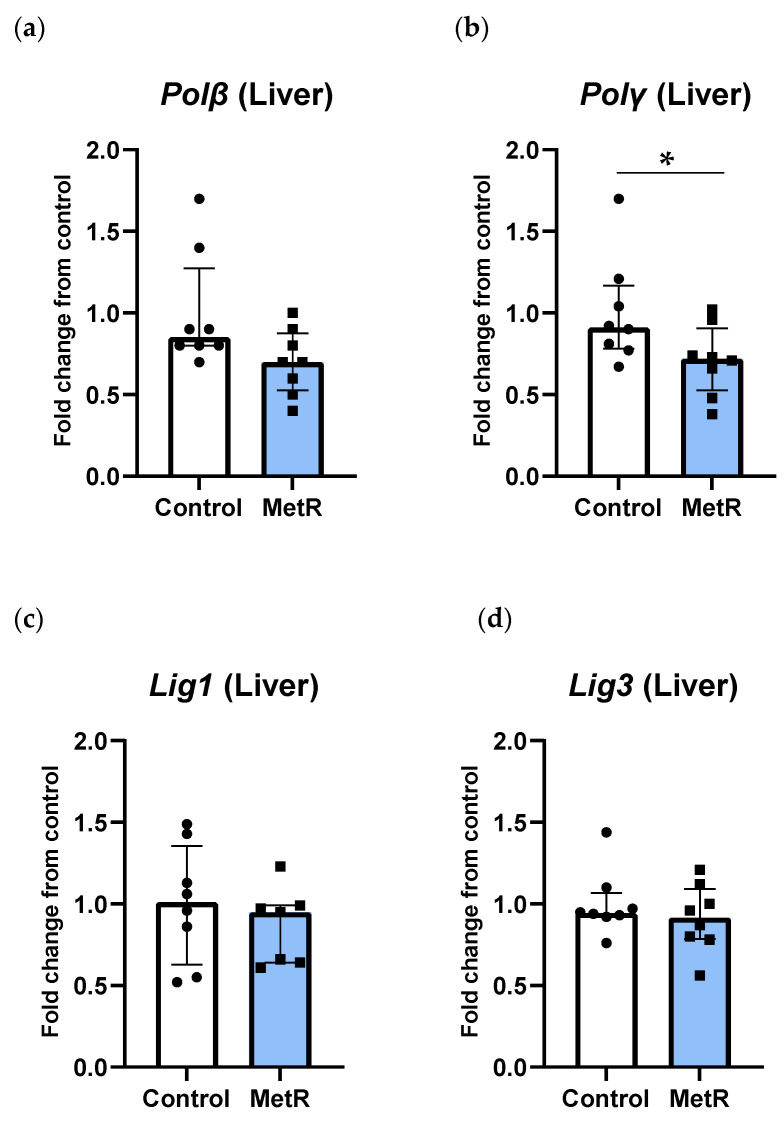
Hepatic mRNA levels of (**a**) *DNA polymerase β*, (**b**) *DNA polymerase γ*, (**c**) *DNA ligase 1*, and (**d**) *DNA ligase 3* of the control (n = 8) and 40% methionine-restricted rats (MetR; n = 8). The results are expressed as the median ± IQR relative to control animals. * *p* ≤ 0.05.

Methionine restriction differently affected DNA polymerase and DNA ligase gene transcription. Transcription of DNA polymerase β and γ genes was reduced after MetR by 30%. However, such reduction reached statistical significance only in *Polγ* (*p* = 0.04) but not in *Polβ* (*p* = 0.06) (Figure 5a,b). In contrast, neither the DNA ligase 1 gene nor the DNA ligase 3 gene hepatic expression changed in the rats subjected to MetR compared to the control animals (*p* ≥ 0.6) (Figure 5c,d).

### 3.5. Ogg1 and Ape1 Genes Were Upregulated in Rat Brain, While Neil2 and Udg Genes Were Not Affected After Methionine Restriction in Rat Brain

**Figure 6:**
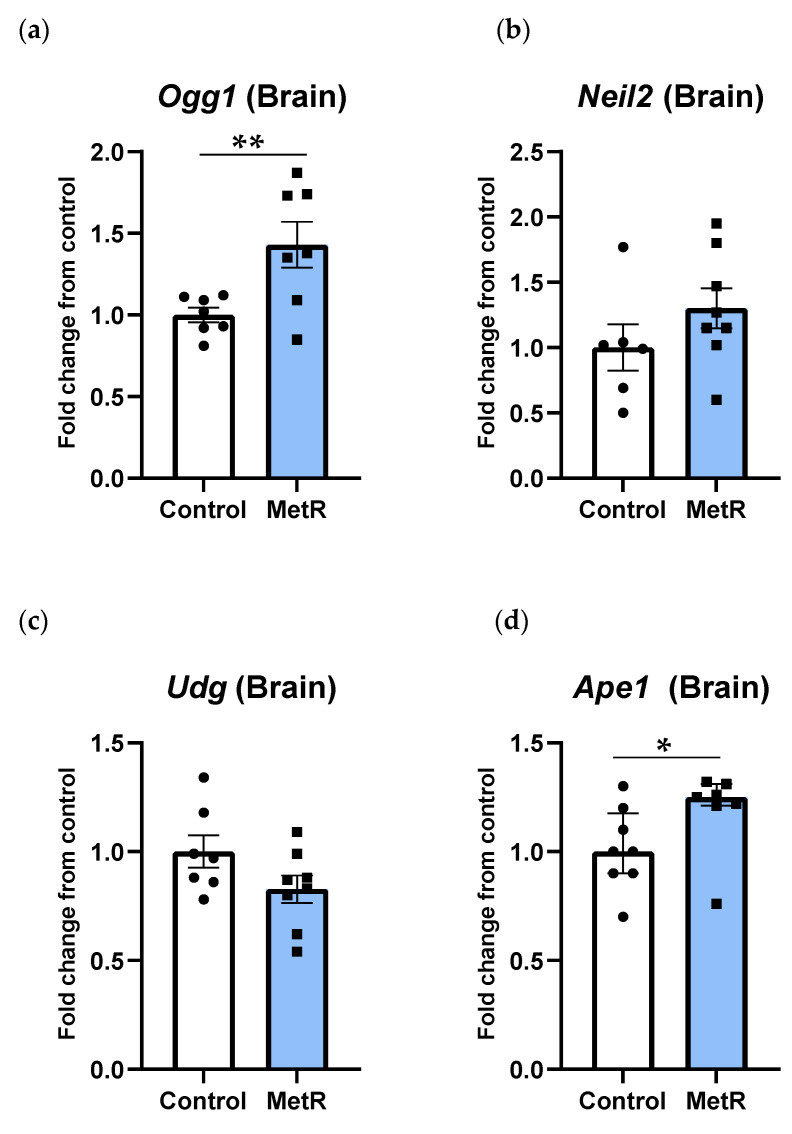
Brain mRNA levels of (**a**) *Ogg1*, (**b**) *Neil2*, (**c**) *Udg*, and (**d**) *Ape1* in the control (n = 7–8) and 40% methionine-restricted rats (MetR; n = 7–8). The results are expressed as the mean ± SEM relative to control animals, except Ape1, which is as the median ± IQR. * *p* ≤ 0.05; ** *p* ≤ 0.01.

In the rat brain, *Ogg1* was the only DNA glycosylase gene whose transcription was affected by MetR (Figure 6a). In contrast to the liver, transcription levels of the *Ogg1* gene were significantly increased by 40% in the brain after MetR (*p* = 0.01). Regarding NEIL2, another DNA glycosylase recognizing and processing oxidative DNA damage, mRNA levels tended to increase in the rat brain after MetR by 30% (Figure 6b), although differences between both experimental groups were not statistically significant (*p* = 0.22). *Udg* gene expression was not modified after MetR in the rat brain (*p* = 0.09) (Figure 6c). Similarly to *Ogg1, Ape1* gene transcription was also significantly upregulated by approximately 15% in brain tissue (*p* = 0.04) (Figure 6d).

### 3.6. Gene Transcription of DNA Polymerases and DNA Ligases Was Differently Modulated by Methionine Restriction in Rat Brain

**Figure 7:**
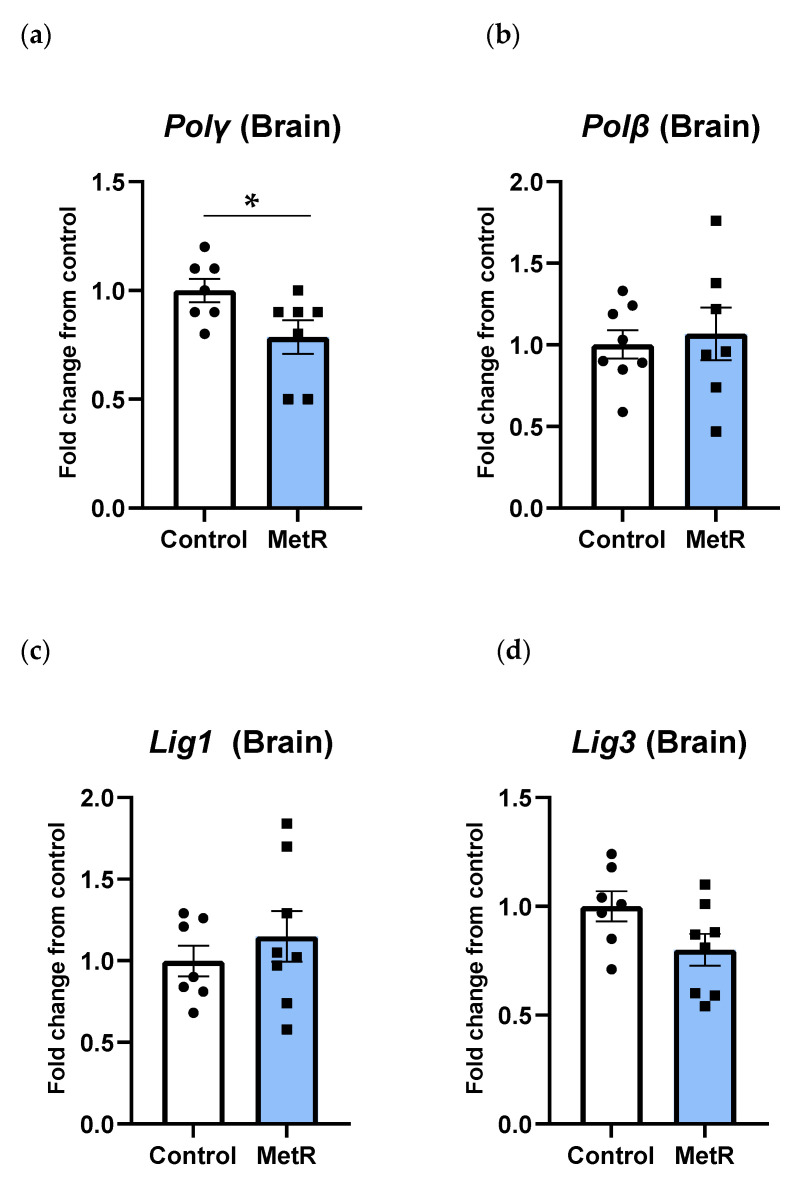
Brain mRNA levels of (**a**) *DNA polymerase γ*, (**b**) *DNA polymerase β*, (**c**) *DNA ligase 1*, and (**d**) *DNA ligase 3* in the control (n = 7–8) and 40% methionine-restricted rats (MetR; n = 7–8). The results are expressed as the mean ± SEM relative to control animals. * *p* ≤ 0.05.

In contrast to the liver, the DNA polymerases β and γ were differently modulated by MetR in the rat brain. Similarly to the liver, a 20% reduction in *Polγ* gene transcription was observed in the rat brain (*p* = 0.04) (Figure 7a); however, no changes were detected in the brain transcription of the *Polβ* gene (*p* = 0.7) (Figure 7b). On the other hand, none of the transcription levels of the two DNA ligases investigated were significantly modified by MetR in the rat brain (Figure 7c,d), despite *Lig3* gene expression being reduced by 20%. However, this decline did not reach statistical significance (*p* = 0.06).

## 4. Discussion

While extensive research has examined the positive impact of a MetR diet on longevity and its association with oxidative stress in rodents (reviewed in [69,70]), it is not known whether the beneficial effect of this dietary manipulation on lifespan might be related to an upregulation in DNA repair as well. In contrast to CR, to our knowledge, no prior studies have investigated the effects of methionine restriction on the BER pathway. Both dietary interventions have been shown to share common biological effects, including reduced mitochondrial ROS production, lower oxidative damage, and extended maximum longevity in rodents [69]. In this study we aimed to determine whether the beneficial effects of a MetR diet in rats are related to changes in the BER pathway in brain and liver, two organs that play a central role in the adaptation to limited methionine intake [70]. Our findings reveal, for the first time, that a 40% methionine restriction in diet increases the expression of *Ogg1* and *Ape1* while decreasing the expression of *Polγ* in the brain. In contrast, MetR decreases the hepatic expression of *Neil2*, *Udg*, and *Ogg1* glycosylases as well as *Ape1* and *Polγ*.

Previous investigations have denoted a complex regulation of BER in animals subjected to CR. For instance, changes in BER capacity after subjecting rats to CR have been shown to be tissue-specific as well as being cellular compartment-dependent in young animals [65]. Moreover, changes in the activity of BER enzymes are not always related to protein levels, but are probably related to post-translational modifications [66,71]. In agreement with the lifespan extension effect of CR, two different studies have reported that CR reverts the age-related decline in the BER pathway in total [66] or nuclear [64] fractions in different rat tissues. Moreover, young calorie-restricted animals showed higher APE1 activity than their control counterparts in different brain regions [66]. Interestingly, in accordance with the cited study, we observed higher *Ape1* gene transcription in the brain cortex of the methionine-restricted animals than in the control ones. In this scenario, and similarly to previous studies, our results also indicate a clear tissue-specific effect of MetR on BER enzyme gene expression. Thus, while we observed a general decline in the transcription levels of BER enzymes in the liver, no changes or enhancements were observed in the brain cortex. However, our results are not entirely comparable with previous ones in CR since ours reflect total gene expression, encompassing both nuclear and mtBER repair; in contrast, previous investigations reported specific BER activities in either nuclear and/or mitochondrial fractions [65]. In addition, as mentioned above, CR and MetR share several physiological effects, but their underlying mechanisms may differ significantly.

**Figure 8:**
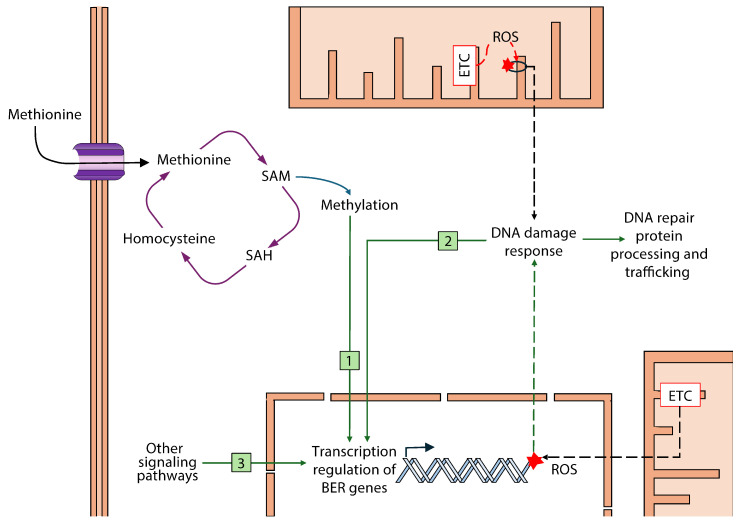
Potential effects of methionine restriction on transcription levels of genes involved in BER. The results in the current investigation suggest that MetR differently affects the gene transcription of BER enzymes in the brain and liver of rats subjected to this dietary intervention. Direct changes in gene transcription due to MetR-associated alterations in DNA methylation processes (1), DNA damage response mechanisms activated after oxidative damage to DNA (2), and other signaling pathways, such as AMPK signaling (3), are likely to be involved in the regulation of BER capacity in cells of those tissues through gene expression modulation of different BER proteins (see the discussion for further details). ETC: electron transport chain; ROS: reactive oxygen species; SAM: S-adenosyl-methionine; SAH: S-adenosyl-homocysteine. Red starts indicate DNA damage. See the discussion for more details.

One potential mechanism involved in the modulation of the BER enzyme gene expression after MetR is DNA methylation (Figure 8). Methylation of DNA is a dynamic process that influences gene transcription and is strongly linked to MetR [72,73]. Specifically, in relation to DNA repair capacity, growing evidence suggests that epigenetic events, including altered DNA methylation, may contribute to the aging process and play a functional role through the dysregulation of the BER enzyme gene expression. Consistent with this, a study conducted in the mouse brain indicates that *Ogg1* and *Neil1* gene expression can be epigenetically regulated, potentially contributing to the effects of aging on DNA repair in the brain [74]. It has been observed that promoters of some BER genes (*Neil1*, *Udg*, and *Ogg1*, among others) may be methylated, resulting in downregulation in gene expression [74]. Regarding methionine intake, it is well established that methionine metabolism directly affects DNA methylation, as methionine serves as the direct precursor of S-adenosylmethionine (SAM) and S-adenosylhomocysteine (SAH), which function as the substrate and product of methylation reactions, respectively [75]. Studies in vitro have shown that physiologically relevant concentrations of methionine altered methylation status (H3K4me3) and ratio of SAM/SAH in cell culture media [73]. In vivo, previous studies have shown that dietary methionine supplementation increases SAM levels, a key methyl donor for DNA methylation, in the liver and heart of rats [76]. Conversely, methionine restriction has been reported to globally reduce DNA methylation levels in the heart [77], whereas other studies have shown an increase in global methylation in the liver and a decline in adipose tissue in adult mice as well as altered methionine cycles and histone methylation [78]. Collectively, these studies provide evidence that MetR can alter epigenetic markers. Therefore, we propose that modifications in DNA methylation patterns following MetR may contribute to BER enzyme gene regulation, potentially altering gene expression patterns. However, further studies are necessary to elucidate how methionine metabolism and histone methylation regulate the transcription of genes involved in maintaining genome stability.

In the brain, MetR has been shown to improve cognitive health by promoting anti-inflammatory transcription factors, such as Nrf2; certain antioxidant pathways, like GSH; or enhancing gene markers of hippocampal plasticity and improving factors related to neuron growth and survival [79]. In this context, it is not surprising that we have found an increase in *Ape1* and *Ogg1* gene expression, reinforcing the idea of its regulation by oxidative stress. APE1 has been proposed as the core BER enzyme, characterized as the rate-limiting step of the process and thus, a critical one. Hence, both APE1 and OGG1 may be considered as key regulators of endogenous DNA damage [80]. In contrast, we have found a decrease in *Polγ* gene expression, the mitochondrial replicative polymerase, that may be due to its different regulation for its unique mitochondrial location and function [81]. In addition, the complexity of the brain implies that BER regulation may vary among brain regions and brain cell types. In fact, various studies have reported that different brain regions present different BER capacity and are differently affected by aging [66,71]. On the other hand, it is important to stress that within the brain, different cell types have been reported to differ in BER capacity [82,83]

In the liver, MetR has a profound effect on lipid and glucose metabolism, resulting in increased insulin sensitivity and reduced risk of diabetes mellitus and hypertension [84,85]. In addition, even a low level of methionine restriction (40%), like they used in this study, decreases oxidative damage to biomolecules [61,77]. In our study, we have observed a decrease in the gene expression of major BER enzymes.

The observed differences in the BER enzyme gene expression between the liver and the brain could be attributed to the organ-specific effects of MetR on oxidative damage. In the liver, MetR has been shown to reduce mtROS production and significantly decrease levels of 8-oxodG in mtDNA [77,86]. In contrast, oxidative damage in mtDNA does not decrease in the brain following MetR [87]. Recent evidence supports a role for DNA damage, particularly in the form of 8-oxoG, as a regulatory mark influencing DNA transcription [88]. Studies in cell cultures have demonstrated that oxidative damage upregulates the gene transcription of major BER enzymes, including *Udg*, *Ogg1*, *Neil2*, and *Ape1* [89,90]. Increasing evidence also suggests that DNA damage response (DDR) signaling influences the transcription of repair genes, particularly of DNA glycosylases. Moreover, the latter also interacts with transcription factors, contributing to DDR modulation of the BER enzyme gene expression. Therefore, the 30–40% reduction in the BER enzyme gene transcription observed in the liver may correspond to the lower oxidative damage in mtDNA, reducing the need for DNA repair and leading to a downregulation of BER capacity. Conversely, in the brain, where oxidative damage in mtDNA remains unchanged after MetR, the upregulation of certain BER genes may represent a compensatory response. Because of their post-mitotic status and relatively low levels of antioxidant defenses, neurons rely heavily on their DNA repair capacity for maintaining DNA integrity. In relation to *Mnsod* expression, we have found lack of changes both in the liver and brain after MetR, in accordance with what has previously been shown regarding SOD protein level in the liver following MetR at old age [77]. In contrast, it has also been shown a decrease in the level of the GSH antioxidant after MetR in the liver [91], while it remained without changes in the brain [92].

The link between oxidative damage, transcription factors involved in BER regulation, and methylation of DNA patterns that alter BER transcription may be elucidated in order to understand a dietary manipulation that is known to have considerable effects on health. Targeting methionine metabolism not only extends lifespan in multiple species, but is also a promising strategy for controlling cancer [93]. Although the regulatory mechanisms that modulate BER enzyme gene expression are not yet fully understood, some proposed mechanisms suggest a potential link to MetR. Among others, AMPK and Sirtuins pathways have been associated with oxidative stress and BER modulation, potentially acting as DNA damage sensors [94,95]. Methionine restriction studies have shown an activation of the AMPK pathway via fibroblast growth factor 21 (FGF21) in the brain [79,96], which could explain the observed increase in *Ogg1* and *Ape1* gene expression. In the liver, a reduction in Sirtuin 1 levels [77] has been shown, which aligns with the generalized reduction in hepatic BER enzyme gene expression observed in our study. However, other sirtuins or transcription factors have also been linked to BER regulation, and future studies should address whether these factors may function as MetR effectors.

Although this study provides valuable insights into the transcriptional BER profile associated with MetR, it is important to note some limitations of the study. On the one hand, our analysis focused on gene expression levels. While this may offer a useful first step in identifying potential regulatory pathways, gene expression may not correlate directly with protein levels or functional enzyme activity. Post-transcriptional and post-translational modifications could significantly influence protein function, leading to discrepancies between gene expression and biological effects. Therefore, future studies should incorporate complementary approaches such as protein level quantification or enzymatic assays to validate and expand our findings. On the other hand, only male rats were used. Previous studies regarding MetR effects on oxidative stress have been mostly made in male subjects, which allows for more direct comparisons. However, this limits the extension of the results to female populations, as biological sex differences may influence the response to methionine restriction. Future studies should aim to include both female and male rodents to provide a more representative understanding of the effects.

## 5. Conclusions

In conclusion, the beneficial effects achieved by MetR are associated with different regulations of the BER gene expression in the liver and brain. We have observed that even a low methionine restriction level (40%) decreased expression of major BER gene enzymes in the liver and increased expression of two limiting and core BER genes, *Ape1* and *Ogg1*, in the brain. This is particularly interesting since MetR may confer a higher degree of DDR (improving BER) in specific brain areas, contributing to better cognitive and neurological maintenance and performance. This regulation may have been mediated by the alteration of DNA methylation patterns and oxidative damage in mtDNA.

## Figures and Tables

**Table 1:** Detailed composition of semi-purified diets used in this study (control and 40% methionine restriction).

Component	Control (g/100 g)	40% MetR (g/100 g)
L-Arginine	1.12	1.124
L-Lysine	1.44	1.445
L-Histidine	0.33	0.331
L-Leucine	1.11	1.114
L-Isoleucine	0.82	0.823
L-Valine	0.82	0.823
L-Threonine	0.82	0.823
L-Tryptophan	0.18	0.181
L-Methionine	0.86	0.516
L-Glutamic acid	2.70	2.709
L-Phenylalanine	1.16	1.164
L-Glycine	2.33	2.338
Dextrine	5	5.017
Corn starch	31.80	31.92
Sucrose	31.79	31.92
Cellulose	5	5.017
Choline bitartrate	0.2	0.201
MP Vitamin diet fortification mixture	1.02	1.023
Mineral mix (AIN)	3.5	3.512
Corn oil	8	8.028
Total (% weight)	100	100

**Table 2:** Primers used in RT-PCR experiments.

*Ogg1*	Forward	TATCATGGCTTCCCAAACCT
	Reverse	CAACTTCTGAGGTGGGTCT
*Udg*	Forward	GATGTGCGACATCCGTGA
	Reverse	TGAGCTTGATTAGGTCCGTGA
*Neil2*	Forward	CAAAAGAGGTGACTGGATAGACC
	Reverse	GGAAGCCACCACCACTAAAA
*Ape1*	Forward	GCATCTGCCACACTCAAG
	Reverse	TGGTGCTTCTTCCTTTACCC
*Polß*	Forward	CAGCGAGAATGATGAAAACG
	Reverse	CTGATCTTTGGGGATCAACC
*Polγ*	Forward	GCGAATGGTCCAGCGATTTC
	Reverse	TTGACGAACAGTTCCCGAGG
*Lig1*	Forward	TGGTGTCCCGCAAAGTGATT
	Reverse	CCTTTGGAGGGGGTTCCTTC
*Lig3*	Forward	TTGCAGAAAGGCTCCACAG
	Reverse	CAAGTTGTACACACTCTTGATGACAC
*Mnsod*	Forward	GCCTCAGCAATGTTGTGTCG
	Reverse	ATTGTTCACGTAGGTCGCGT
*Ppargc1a*	Forward	TTCAGGAGCTGGATGGCTTG
	Reverse	GGGCAGCACACTCTATGTCA
*Bdnf*	Forward	TGTTGGGGAGACGAGATTTT
	Reverse	TCACCTGGTGGAACATTGTG
*18S*	Forward	GGTGCATGGCCGTTCTTA
	Reverse	TCGTTCGTTATCGGAATTAACC

## Data Availability

The original contributions presented in the study are included in the article. Further inquiries can be directed to the corresponding authors.

## References

[1] Lopez-Otín C., Blasco M.A., Partridge L., Serrano M., Kroemer G. (2013). The hallmarks of aging. Cell.

[2] Lopez-Otín C., Blasco M.A., Partridge L., Serrano M., Kroemer G. (2023). Hallmarks of aging: An expanding universe. Cell.

[3] Willcox D.C., Scapagnini G., Willcox B.J. (2014). Healthy aging diets other than the Mediterranean: A focus on the Okinawan diet. Mech. Ageing Dev..

[4] McCay C.M., Crowell M.F., Maynard L.A. (1935). The Effect of Retarded Growth Upon the Length of Life Span and Upon the Ultimate Body Size: One Figure. J. Nutr..

[5] Weindruch R. (1996). The retardation of aging by caloric restriction: Studies in rodents and primates. Toxicol. Pathol..

[6] Wu Z., Qu J., Liu G.-H. (2024). Roles of chromatin and genome instability in cellular senescence and their relevance to ageing and related diseases. Nat. Rev. Mol. Cell Biol..

[7] Ropert B., Gallrein C., Schumacher B. (2024). DNA repair deficiencies and neurodegeneration. DNA Repair.

[8] Akbari M., Kirkwood T.B., Bohr V.A. (2019). Mitochondria in the signaling pathways that control longevity and health span. Ageing Res. Rev..

[9] Gredilla R., Bohr V.A., Stevnsner T. (2010). Mitochondrial DNA repair and association with aging—An update. Exp. Gerontol..

[10] Jeppesen D.K., Bohr V.A., Stevnsner T. (2011). DNA repair deficiency in neurodegeneration. Prog. Neurobiol..

[11] Christmann M., Tomicic M.T., Roos W.P., Kaina B. (2003). Mechanisms of human DNA repair: An update. Toxicology.

[12] de Souza-Pinto N.C., Mason P.A., Hashiguchi K., Weissman L., Tian J., Guay D., Lebel M., Stevnsner T.V., Rasmussen L.J., Bohr V.A. (2009). Novel DNA mismatch-repair activity involving YB-1 in human mitochondria. DNA Repair.

[13] Aamann M.D., Sorensen M.M., Hvitby C., Berquist B.R., Muftuoglu M., Tian J., de Souza-Pinto N.C., Scheibye-Knudsen M., Wilson D.M., Stevnsner T. (2010). Cockayne syndrome group B protein promotes mitochondrial DNA stability by supporting the DNA repair association with the mitochondrial membrane. FASEB J..

[14] Krokan E.H., Nilsen H., Skorpen F., Otterlei M., Slupphaug G. (2000). Base excision repair of DNA in mammalian cells. FEBS Lett..

[15] Chattopadhyay R., Wiederhold L., Szczesny B., Boldogh I., Hazra T.K., Izumi T., Mitra S. (2006). Identification and characterization of mitochondrial abasic (AP)-endonuclease in mammalian cells. Nucleic Acids Res..

[16] Dianov G.L., Souza-Pinto N., Nyaga S.G., Thybo T., Stevnsner T., Bohr V.A. (2001). Base excision repair in nuclear and mitochondrial DNA. Prog. Nucleic Acid Res. Mol. Biol..

[17] Bohr V.A. (2002). Repair of oxidative DNA damage in nuclear and mitochondrial DNA, and some changes with aging in mammalian cells. Free. Radic. Biol. Med..

[18] Behrouzi A., Kelley M.R., Fehrenbacher J.C. (2022). Oxidative DNA Damage: A Role in Altering Neuronal Function. J. Cell. Signal..

[19] Krasich R., Copeland W.C. (2017). DNA polymerases in the mitochondria: A critical review of the evidence. Front. Biosci..

[20] Zárate S., Stevnsner T., Gredilla R. (2017). Role of Estrogen and Other Sex Hormones in Brain Aging. Neuroprotection and DNA Repair. Front. Aging Neurosci..

[21] Kim Y.J., Kim H.S., Seo Y.R., Maluf S.W. (2018). Genomic Approach to Understand the Association of DNA Repair with Longevity and Healthy Aging Using Genomic Databases of Oldest-Old Population. Oxidat. Med. Cell. Longev..

[22] Garagnani P., Marquis J., Delledonne M., Pirazzini C., Marasco E., Kwiatkowska K.M., Iannuzzi V., Bacalini M.G., Valsesia A., Carayol J. (2021). Whole-genome sequencing analysis of semi-supercentenarians. eLife.

[23] Maynard S., Fang E.F., Scheibye-Knudsen M., Croteau D.L., Bohr V.A. (2015). DNA Damage, DNA Repair, Aging, and Neurodegeneration. Cold Spring Harb. Perspect. Med..

[24] Lautrup S., Hou Y., Fang E.F., Bohr V.A. (2024). Roles of NAD^+^ in Health and Aging. Cold Spring Harb. Perspect. Med..

[25] Fang E.F., Lautrup S., Hou Y., Demarest T.G., Croteau D.L., Mattson M.P., Bohr V.A. (2017). NAD^+^ in Aging: Molecular Mechanisms and Translational Implications. Trends Mol. Med..

[26] Hou Y., Lautrup S., Cordonnier S., Wang Y., Croteau D.L., Zavala E., Zhang Y., Moritoh K., O’connell J.F., Baptiste B.A. (2018). NAD^+^ supplementation normalizes key Alzheimer’s features and DNA damage responses in a new AD mouse model with introduced DNA repair deficiency. Proc. Natl. Acad. Sci. USA.

[27] Fang E.F., Kassahun H., Croteau D.L., Scheibye-Knudsen M., Marosi K., Lu H., Shamanna R.A., Kalyanasundaram S., Bollineni R.C., Wilson M.A. (2016). NAD^+^ Replenishment Improves Lifespan and Healthspan in Ataxia Telangiectasia Models via Mitophagy and DNA Repair. Cell Metab..

[28] Weindruch R., Walford R.L., Fligiel S., Guthrie D. (1986). The retardation of aging in mice by dietary restriction: Longevity, cancer, immunity and lifetime energy intake. J. Nutr..

[29] Green C.L., Lamming D.W., Fontana L. (2022). Molecular mechanisms of dietary restriction promoting health and longevity. Nat. Rev. Mol. Cell Biol..

[30] Dhahbi J.M., Kim H.-J., Mote P.L., Beaver R.J., Spindler S.R. (2004). Temporal linkage between the phenotypic and genomic responses to caloric restriction. Proc. Natl. Acad. Sci. USA.

[31] Lee C.-K., Allison D.B., Brand J., Weindruch R., Prolla T.A. (2002). Transcriptional profiles associated with aging and middle age-onset caloric restriction in mouse hearts. Proc. Natl. Acad. Sci. USA.

[32] Yu B.P., Masoro E.J., McMahan C.A. (1985). Nutritional influences on aging of Fischer 344 rats: I. Physical, metabolic, and longevity characteristics. J. Gerontol..

[33] Martin B., Mattson M.P., Maudsley S. (2006). Caloric restriction and intermittent fasting: Two potential diets for successful brain aging. Ageing Res. Rev..

[34] Mattson M.P., Chan S.L., Duan W. (2002). Modification of brain aging and neurodegenerative disorders by genes, diet, and behavior. Physiol. Rev..

[35] Weindruch R. (2003). Caloric restriction, gene expression, and aging. Alzheimer Dis. Assoc. Disord..

[36] Colman R.J., Anderson R.M., Johnson S.C., Kastman E.K., Kosmatka K.J., Beasley T.M., Allison D.B., Cruzen C., Simmons H.A., Kemnitz J.W. (2009). Caloric restriction delays disease onset and mortality in rhesus monkeys. Science.

[37] Colman R.J., Beasley T.M., Kemnitz J.W., Johnson S.C., Weindruch R., Anderson R.M. (2014). Caloric restriction reduces age-related and all-cause mortality in rhesus monkeys. Nat. Commun..

[38] Fontana L., Klein S., Holloszy J.O. (2010). Effects of long-term calorie restriction and endurance exercise on glucose tolerance, insulin action, and adipokine production. AGE.

[39] Sohal R.S., Weindruch R. (1996). Oxidative stress, caloric restriction, and aging. Science.

[40] Gredilla R., Barja G., López-Torres M. (2001). Effect of short-term caloric restriction on H_2_O_2_ production and oxidative DNA damage in rat liver mitochondria and location of the free radical source. J. Bioenerg. Biomembr..

[41] Gredilla R., Sanz A., Lopez-Torres M., Barja G. (2001). Caloric restriction decreases mitochondrial free radical generation at complex I and lowers oxidative damage to mitochondrial DNA in the rat heart. FASEB J..

[42] López-Torres M., Gredilla R., Sanz A., Barja G. (2002). Influence of aging and long-term caloric restriction on oxygen radical generation and oxidative DNA damage in rat liver mitochondria. Free. Radic. Biol. Med..

[43] Bevilacqua L., Ramsey J.J., Hagopian K., Weindruch R., Harper M.-E. (2004). Effects of short- and medium-term calorie restriction on muscle mitochondrial proton leak and reactive oxygen species production. Am. J. Physiol. Endocrinol. Metab..

[44] Sanz A., Caro P., Ibañez J., Gómez J., Gredilla R., Barja G. (2005). Dietary restriction at old age lowers mitochondrial oxygen radical production and leak at complex I and oxidative DNA damage in rat brain. J. Bioenerg. Biomembr..

[45] Sohal R.S., Agarwal S., Candas M., Forster M.J., Lal H. (1994). Effect of age and caloric restriction on DNA oxidative damage in different tissues of C57BL/6 mice. Mech. Ageing Dev..

[46] Hagopian K., Chen Y., Domer K.S., Hoo R.S., Bentley T., McDonald R.B., Ramsey J.J. (2011). Caloric restriction influences hydrogen peroxide generation in mitochondrial sub-populations from mouse liver. J. Bioenerg. Biomembr..

[47] Mair W., Piper M.D.W., Partridge L., Kirkwood T. (2005). Calories do not explain extension of life span by dietary restriction in Drosophila. PLoS Biol..

[48] Austad S.N., Hoffman J.M. (2021). Beyond calorie restriction: Aging as a biological target for nutrient therapies. Curr. Opin. Biotechnol..

[49] López-Torres M., Barja G. (2008). Lowered methionine ingestion as responsible for the decrease in rodent mitochondrial oxidative stress in protein and dietary restriction: Possible implications for humans. Biochim. Biophys. Acta.

[50] Orentreich N., Matias J.R., DeFelice A., Zimmerman J.A. (1993). Low methionine ingestion by rats extends life span. J. Nutr..

[51] Richie J.P., Leutzinger Y., Parthasarathy S., Maixoy V., Orentreich N., Zimmerman J.A. (1994). Methionine restriction increases blood glutathione and longevity in F344 rats. FASEB J..

[52] Miller R.A., Buehner G., Chang Y., Harper J.M., Sigler R., Smith-Wheelock M. (2005). Methionine-deficient diet extends mouse lifespan, slows immune and lens aging, alters glucose, T4, IGF-I and insulin levels, and increases hepatocyte MIF levels and stress resistance. Aging Cell.

[53] Sun L., Akha A.A.S., Miller R.A., Harper J.M. (2009). Life-span extension in mice by preweaning food restriction and by methionine restriction in middle age. J. Gerontol. Ser. A Biol. Sci. Med. Sci..

[54] Petti A.A., Crutchfield C.A., Rabinowitz J.D., Botstein D. (2011). Survival of starving yeast is correlated with oxidative stress response and nonrespiratory mitochondrial function. Proc. Natl. Acad. Sci. USA.

[55] Lee B.C., Kaya A., Ma S., Kim G., Gerashchenko M.V., Yim S.H., Hu Z., Harshman L.G., Gladyshev V.N. (2014). Methionine restriction extends lifespan of Drosophila melanogaster under conditions of low amino-acid status. Nat. Commun..

[56] Cabreiro F., Au C., Leung K.-Y., Vergara-Irigaray N., Cochemé H.M., Noori T., Weinkove D., Schuster E., Greene N.D., Gems D. (2013). Metformin retards aging in *C. elegans* by altering microbial folate and methionine metabolism. Cell.

[57] Malloy V.L., Krajcik R.A., Bailey S.J., Hristopoulos G., Plummer J.D., Orentreich N. (2006). Methionine restriction decreases visceral fat mass and preserves insulin action in aging male Fischer 344 rats independent of energy restriction. Aging Cell.

[58] Zhang Y., Jelleschitz J., Grune T., Chen W., Zhao Y., Jia M., Wang Y., Liu Z., Höhn A. (2022). Methionine restriction—Association with redox homeostasis and implications on aging and diseases. Redox Biol..

[59] Wang C., Hei Y., Liu Y., Bajpai A.K., Li Y., Guan Y., Xu F., Yao C. (2024). Systems genetics identifies methionine as a high risk factor for Alzheimer’s disease. Front. Neurosci..

[60] Sanz A., Caro P., Ayala V., Portero-Otin M., Pamplona R., Barja G. (2006). Methionine restriction decreases mitochondrial oxygen radical generation and leak as well as oxidative damage to mitochondrial DNA and proteins. FASEB J..

[61] Sanchez-Roman I., Gomez A., Gomez J., Suarez H., Sanchez C., Naudi A., Ayala V., Portero-Otin M., Lopez-Torres M., Pamplona R. (2011). Forty percent methionine restriction lowers DNA methylation, complex I ROS generation, and oxidative damage to mtDNA and mitochondrial proteins in rat heart. J. Bioenerg. Biomembr..

[62] Most J., Tosti V., Redman L.M., Fontana L. (2017). Calorie restriction in humans: An update. Ageing Res. Rev..

[63] Racette S.B., Weiss E.P., Villareal D.T., Arif H., Steger-May K., Schechtman K.B., Fontana L., Klein S., Holloszy J.O., The Washington University School of Medicine CALERIE Group (2006). One year of caloric restriction in humans: Feasibility and effects on body composition and abdominal adipose tissue. J. Gerontol. Ser. A Biol. Sci. Med. Sci..

[64] Cabelof D. (2003). Caloric restriction promotes genomic stability by induction of base excision repair and reversal of its age-related decline. DNA Repair.

[65] Stuart J.A., Karahalil B., Hogue B.A., Souza-Pinto N.C., Bohr V.A. (2004). Mitochondrial and nuclear DNA base excision repair are affected differently by caloric restriction. FASEB J..

[66] Kisby G.E., Kohama S.G., Olivas A., Churchwell M., Doerge D., Spangler E., de Cabo R., Ingram D.K., Imhof B., Bao G. (2009). Effect of caloric restriction on base-excision repair (BER) in the aging rat brain. Exp. Gerontol..

[67] Hunt N.J., Kang S.W.S., Lockwood G.P., Le Couteur D.G., Cogger V.C. (2019). Hallmarks of Aging in the Liver. Comput. Struct. Biotechnol. J..

[68] Li Q., Xiao N., Zhang H., Liang G., Lin Y., Qian Z., Yang X., Yang J., Fu Y., Zhang C. (2025). Systemic aging and aging-related diseases. FASEB J..

[69] Sanchez-Roman I., Barja G. (2013). Regulation of longevity and oxidative stress by nutritional interventions: Role of methionine restriction. Exp. Gerontol..

[70] Mladenović D., Radosavljević T., Hrnčić D., Rasic-Markovic A., Stanojlović O. (2019). The effects of dietary methionine restriction on the function and metabolic reprogramming in the liver and brain—Implications for longevity. Prog. Neurobiol..

[71] Gredilla R., Garm C., Holm R., Bohr V.A., Stevnsner T. (2010). Differential age-related changes in mitochondrial DNA repair activities in mouse brain regions. Neurobiol. Aging.

[72] Zhang N. (2018). Role of methionine on epigenetic modification of DNA methylation and gene expression in animals. Anim. Nutr..

[73] Dai Z., Mentch S.J., Gao X., Nichenametla S.N., Locasale J.W. (2018). Methionine metabolism influences genomic architecture and gene expression through H3K4me3 peak width. Nat. Commun..

[74] Langie S.A.S., Cameron K.M., Ficz G., Oxley D., Tomaszewski B., Gorniak J.P., Maas L.M., Godschalk R.W.L., Van Schooten F.J., Reik W. (2017). The Ageing Brain: Effects on DNA Repair and DNA Methylation in Mice. Genes.

[75] Parkhitko A.A., Jouandin P., Mohr S.E., Perrimon N. (2019). Methionine metabolism and methyltransferases in the regulation of aging and lifespan extension across species. Aging Cell.

[76] Gomez J., Caro P., Sanchez I., Naudi A., Jove M., Portero-Otin M., Lopez-Torres M., Pamplona R., Barja G. (2009). Effect of methionine dietary supplementation on mitochondrial oxygen radical generation and oxidative DNA damage in rat liver and heart. J. Bioenerg. Biomembr..

[77] Sanchez-Roman I., Gómez A., Pérez I., Sanchez C., Suarez H., Naudí A., Jové M., Lopez-Torres M., Pamplona R., Barja G. (2012). Effects of aging and methionine restriction applied at old age on ROS generation and oxidative damage in rat liver mitochondria. Biogerontology.

[78] Mattocks D.A., Mentch S.J., Shneyder J., Ables G.P., Sun D., Richie J.P., Locasale J.W., Nichenametla S.N. (2017). Short term methionine restriction increases hepatic global DNA methylation in adult but not young male C57BL/6J mice. Exp. Gerontol..

[79] Lail H., Mabb A.M., Parent M.B., Pinheiro F., Wanders D. (2023). Effects of Dietary Methionine Restriction on Cognition in Mice. Nutrients.

[80] Dyrkheeva N.S., Lebedeva N.A., Lavrik O.I. (2016). AP Endonuclease 1 as a Key Enzyme in Repair of Apurinic/Apyrimidinic Sites. Biochemistry.

[81] Kaguni L.S. (2004). DNA polymerase gamma, the mitochondrial replicase. Annu. Rev. Biochem..

[82] Fishel M.L., Vasko M.R., Kelley M.R. (2007). DNA repair in neurons: So if they don’t divide what’s to repair?. Mutat. Res..

[83] Hollensworth S.B., Shen C.-C., Sim J.E., Spitz D.R., Wilson G.L., LeDoux S.P. (2000). Glial cell type-specific responses to menadione-induced oxidative stress. Free. Radic. Biol. Med..

[84] Ables G.P., Perrone C.E., Orentreich D., Orentreich N., Borras C. (2012). Methionine-restricted C57BL/6J mice are resistant to diet-induced obesity and insulin resistance but have low bone density. PLoS ONE.

[85] Perrone C.E., Malloy V.L., Orentreich D.S., Orentreich N. (2013). Metabolic adaptations to methionine restriction that benefit health and lifespan in rodents. Exp. Gerontol..

[86] Caro P., Gómez J., López-Torres M., Sánchez I., Naudí A., Jove M., Pamplona R., Barja G. (2008). Forty percent and eighty percent methionine restriction decrease mitochondrial ROS generation and oxidative stress in rat liver. Biogerontology.

[87] Caro P., Gomez J., Sanchez I., Naudi A., Ayala V., López-Torres M., Pamplona R., Barja G. (2009). Forty percent methionine restriction decreases mitochondrial oxygen radical production and leak at complex I during forward electron flow and lowers oxidative damage to proteins and mitochondrial DNA in rat kidney and brain mitochondria. Rejuvenation Res..

[88] Bordin D.L., Lirussi L., Nilsen H. (2021). Cellular response to endogenous DNA damage: DNA base modifications in gene expression regulation. DNA Repair.

[89] Akbari M., Otterlei M., Peña-Diaz J., Krokan H. (2007). Different organization of base excision repair of uracil in DNA in nuclei and mitochondria and selective upregulation of mitochondrial uracil-DNA glycosylase after oxidative stress. Neuroscience.

[90] Holst C.M., Andersen N.B., Thinggaard V., Tilken M., Lautrup S., Tesauro C., Stevnsner T. (2023). Phosphorylation of the Human DNA Glycosylase NEIL2 Is Affected by Oxidative Stress and Modulates Its Activity. Antioxidants.

[91] Maddineni S., Nichenametla S., Sinha R., Wilson R.P., Richie J.P. (2013). Methionine restriction affects oxidative stress and glutathione-related redox pathways in the rat. Exp. Biol. Med..

[92] Richie J.P., Komninou D., Leutzinger Y., Kleinman W., Orentreich N., Malloy V., Zimmerman J.A. (2004). Tissue glutathione and cysteine levels in methionine-restricted rats. Nutrition.

[93] Wang Z., Yip L.Y., Lee J.H.J., Wu Z., Chew H.Y., Chong P.K.W., Teo C.C., Ang H.Y.-K., Peh K.L.E., Yuan J. (2019). Methionine is a metabolic dependency of tumor-initiating cells. Nat. Med..

[94] Szewczuk M., Boguszewska K., Kaźmierczak-Barańska J., Karwowski B.T. (2020). The role of AMPK in metabolism and its influence on DNA damage repair. Mol. Biol. Rep..

[95] Geng A., Sun J., Tang H., Yu Y., Wang X., Zhang J., Wang X., Sun X., Zhou X., Gao N. (2024). SIRT2 promotes base excision repair by transcriptionally activating OGG1 in an ATM/ATR-dependent manner. Nucleic Acids Res..

[96] Zhang Y., Wang Y., Li Y., Pang J., Höhn A., Dong W., Gao R., Liu Y., Wang D., She Y. (2024). Methionine restriction alleviates diabetes-associated cognitive impairment via activation of FGF21. Redox Biol..

